# KRAS Mutation Status in Relation to Clinicopathological Characteristics of Romanian Colorectal Cancer Patients

**DOI:** 10.3390/cimb47020120

**Published:** 2025-02-12

**Authors:** Elena-Roxana Avădănei, Irina-Draga Căruntu, Irina Nucă, Raluca Anca Balan, Ludmila Lozneanu, Simona-Eliza Giusca, Diana Lavinia Pricope, Cristina Gena Dascalu, Cornelia Amalinei

**Affiliations:** 1 Department of Morpho-Functional Sciences I-Histology, Pathology, “Grigore T. Popa” University of Medicine and Pharmacy, 16 University Street, 700115 Iasi, Romania; irina.caruntu@umfiasi.ro (I.-D.C.); raluca.balan@umfiasi.ro (R.A.B.); ludmila.lozneanu@umfiasi.ro (L.L.); simonaelizagiusca@gmail.com (S.-E.G.); dianalavinia64@gmail.com (D.L.P.); cornelia.amalinei@umfiasi.ro (C.A.); 2 Praxis Medical Investigation Laboratory, 35 Moara de Vant Street, 700376 Iasi, Romania; irina.resmerita@umfiasi.ro; 3 Romanian Medical Science Academy, 1 I.C. Bratianu Boulevard, 030171 Bucharest, Romania; 4 Department of Mother and Child Medicine-Genetics, “Grigore T. Popa” University of Medicine and Pharmacy, 16 University Street, 700115 Iasi, Romania; 5 Department of Pathology, “Sf. Spiridon” Clinical Emergency County Hospital, 1 Independentei Street, 700111 Iasi, Romania; 6 Department of Preventive Medicine and Interdisciplinarity-Medical Informatics and Biostatistics, “Grigore T. Popa” University of Medicine and Pharmacy, 16 University Street, 700115 Iasi, Romania; cristina.dascalu@umfiasi.ro; 7 Department of Histopathology, Institute of Legal Medicine, 4 Buna Vestire Street, 700455 Iasi, Romania

**Keywords:** *KRAS* mutations, colorectal cancer, clinicopathological features

## Abstract

Our study’s aim was to evaluate the clinicopathological profile of colorectal cancer (CRC) patients from North-East Romania in relation to the Kirsten rat sarcoma viral oncogene homolog (*KRAS*). We designed a retrospective study on 108 CRC patients using the fully automated real-time PCR-based molecular testing system, Idylla^TM^ *KRAS* Mutation Test (Biocartis, Mechelen, Belgium). Of the patients, 64 (59.3%) were men and 62 (57.4%) were older than the group average, with left bowel location in 38 cases (35.2%), adenocarcinoma NOS in 102 cases (94.4%), mixed histological pattern in 65 cases (60.2%), T3 in 60 patients (55.6%), N2 in 46 patients (42.6%), and 7–12 tumour buds registered in 58 tumours (53.7%). A total of 54 tumour samples (50%) showed *KRAS* mutation. Statistical comparative analyses associated *KRAS* mutations with the histopathological pattern (*p* = 0.018), tumour grade (*p* = 0.030), depth of invasion (pT) (*p* < 0.001), lymph node involvement (pN) (*p* < 0.001), venous vascular invasion (*p* = 0.048), and tumour buds’ number (*p* = 0.007). Our results demonstrate the relationship between *KRAS* mutation and clinicopathological features, with possible impact in clinical tumour stratification and therapeutic management.

## 1. Introduction

According to GLOBOCAN 2022 data, colorectal cancer (CRC) is the third most commonly diagnosed cancer in men, with about 8000 new cases per year; the second in women, with more than 5500 new cases per year, in Romania [1]; and the first in both genders in terms of prevalence [1]. About 20–25% of CRC cases have metastatic disease at the time of diagnosis [2,3], and up to 50% of patients with initial local disease develop metastases [4]. Preneoplastic lesions undergo gradual accumulation of genetic and epigenetic changes that inactivate suppressor genes, such as *TP53*, adenomatous polyposis coli (*APC*), and *SMAD* family member *SMAD* 4 (*SMAD4*) and activate tumour pro-oncogenes, such as the Kirsten rat sarcoma viral oncogene homolog (*KRAS*) and phosphatidylinositol-4,5-bisphosphate 3-kinase catalytic subunit alpha (PIK3CA), that provide selective proliferation advantages and induce CRC progression [5]. *KRAS* and neuroblastoma rat sarcoma viral oncogene homolog (*NRAS*) mutations occur early in colorectal tumorigenesis [6]. KRAS and NRAS encode protein guanosine triphosphatases (GTPases), members of the small GTPase superfamily responsible for mitogen-activated protein kinase (MAPK) and phosphoinositide-3 kinase (PI3K) pathways signalling, being involved in the regulation of cell growth, survival, and differentiation [7]. *KRAS* mutations occur in 40–60% of CRC patients [8,9], with the most frequent mutations occurring in codons 12 and 13 (90%), followed by codons 146, 117, 61, and 59 of the KRAS gene [10,11].

Activating *KRAS* mutations are produced by single substitutions of amino acids, and the resulting mutant proteins are associated with variable tumours, such as colorectal carcinoma, pancreatic ductal carcinoma, lung adenocarcinoma, and cholangiocarcinoma [7]. The two *K-Ras* isoforms with different C-terminal regions may be produced by alternative splicing [12]. In addition to missense mutations in three hotspots, misregulation of isoform expression is a cancer-driving factor [13]. Moreover, *KRAS* mutations have been linked to poor outcomes in non-small cell lung cancer and in colorectal cancer [14].

The epidermal growth factor receptor antigen (EGFR), a tyrosine kinase of the ErbB receptor family involved in cell proliferation, is now considered an important therapeutic target in metastatic CRC (mCRC) patients [4]. A key player in the EGFR-activated signalling cascade is KRAS oncoprotein [15]. *KRAS* mutation leads to constitutive alteration of EGFR downstream signalling and induces resistance to anti-EGFR therapy [15]. Thus, *RAS* mutations are regarded as negative predictive markers for treatment with monoclonal antibodies (MoAbs) targeting EGFR [4,16]. The characteristic features of CRCs harbouring *KRAS* mutations are that they occur more likely in men and show classic adenocarcinoma morphology, with differentiated histological pattern and microsatellite stability [17].

Due to their critical role in carcinogenesis, RAS proteins are potential therapeutic targets [18]. The targeting of G12C-mutant KRAS with covalent KRAS-G12C inhibitors adagrasib and sotorasib has certified that direct KRAS targeting may be considered one of the most promising strategies for CRC treatment [19].

Tumour extension staging (TNM) remains the most important prognostic factor used in the therapeutic algorithm [20]. However, genetic alterations associated with disease progression are also responsible for the differences in response to therapy and the prognosis of patients with the same tumour stage [21], demonstrating the necessity to identify new molecular biomarkers that accurately reflect the tumour progression and prognosis [21].

In this context, information regarding the possible associations of demographical, clinical, and pathological characteristics, with the molecular substrate of CRC, would lead to an improved therapeutic management. Moreover, about 15% of CRC patients also show a distinct molecular phenotype, known as microsatellite instability (MSI) [22], in addition to *KRAS*, *NRAS*, and *BRAF* gene mutations. As a consequence, this spectrum of mutations may lead to a specific approach, with anti-KRAS agents included in the therapeutic algorithm [23], contributing to an improved prognosis. However, the increased variability of *RAS* gene mutations leads to difficulties in tailored therapy [24]. Moreover, while tumours with wild-type *RAS* genes are highly sensitive to anti-EGFR therapy, the administration of cetuximab or panitumumab to patients with *RAS* mutations can facilitate tumour growth [25,26]. The choice between anti-EGFR or anti-VEGF therapy is determined by the location of the primary tumour, in patients with RAS wild-type tumours. Accordingly, anti-EGFR therapy is the treatment of choice for left-sided colon tumours, while anti-VEGF therapy is more effective for right-sided colon tumours [11,27].

Considering that molecular genetic testing has become an integral part of CRC management, mCRC patients should be tested for a wide range of mutations, including *KRAS*, *NRAS*, *BRAF*, MSI, and *HER2* [11,28,29]. It is also known that colorectal tumours with *BRAF* gene mutations, specifically V600E mutation, have an unfavourable prognosis but may partially respond to treatment with BRAF inhibitors and anti-EGFR antibodies [30].

According to our knowledge, there are only few studies of *RAS* gene mutations in Romanian CRC patients [31,32,33,34,35,36]. These studies have used different techniques and selection criteria for the group of patients, which consisted of a rather reduced number of cases, ranging from 56 to 372 [31,32,33,34,35,36], resulting in difficult interpretation of mutations occurrence. In this context, our aim is to provide a demographic profile of the patients included in the study group, using a modern technique, associated with CRC histopathological analysis according to current criteria [20,37]. Furthermore, the objective of the study was to achieve a molecular classification according to *KRAS* mutational status and its prognosis value. Considering that *RAS* mutations status is a useful tool in mCRC patients’ treatment personalization, our study is evaluating the possibility to select the potential candidates for anti-EGFR therapy.

## 2. Materials and Methods

### 2.1. Study Group

The study group consisted of 108 patients diagnosed with CRC in the pathology departments of seven hospitals in North-East Romania who have been referred for genetic testing in Praxis Medical Investigations Laboratory of Iasi. Data have been collected from consecutive unselected samples genetically tested in this laboratory from 1 January 2022 to 31 December 2023.

Informed consent for biological material use was obtained from all patients. All procedures were carried out according to the ethical standards of the Declaration of Helsinki. We conducted our research with the approval and in accordance with the guidelines of the local ethics committee (approval no. 135A/11/06/2024).

### 2.2. Histopathological Examination

All cases of the study group were re-evaluated and underwent standardized histopathological examination. Tumours were histologically reassessed, by two independent pathologists (E.R.A. and S.E.G.), using the following morphological parameters: histological type, proportion of tumour exhibiting mucinous differentiation, tumour grade, compressive or infiltrative invasion front type, and tumour budding (TB). Mucinous differentiation was defined by the presence of extracellular mucin and clusters or individual mucinous tumour cells, including signet ring type. The tumour was classified as mucinous carcinoma if >50% of the tumour showed mucinous differentiation. The degree of tumour extension (T), the number of metastatic lymph nodes (N), the presence of distant metastases (M), as well as the identification of vascular emboli (venous and lymphatic vascular invasion) and of perineural invasion were also registered. The histological classification was performed according to the most recent World Health Organization (WHO) criteria [38]. Conventional adenocarcinoma was considered as CRC adenocarcinoma NOS, with tumours classified as low-grade in well-differentiated and moderately differentiated adenocarcinomas when ≥50% of the tumour had a glandular pattern or high-grade in poorly differentiated adenocarcinomas when the tumour had a glandular component of <50%.

Following the histopathological examination, a standard haematoxylin and eosin (H&E) slide was used for each case for the quantification of the percentage of tumour cells of the total tumour volume. According to the standardized protocol [39], the ratio of tumour cells was calculated by nuclei counting in five high-power fields (HPFs) to obtain the percentage of tumour cells. Based on this quantification, paraffin block sections of different thicknesses were taken for genetic samples, as follows: (1) a section of 5 µm thickness if the analysed sample contained ≥10% tumour cells in the formalin-fixed paraffin-embedded (FFPE) tissue section, corresponding to an area ≥50 mm^2^, or (2) a section of 10µm thickness (macrodissection) if the area was ≥25 mm^2^ and the analysed sample contained <10% tumour cells in the FFPE tissue section.

### 2.3. Genetic Testing

An Idylla™ RAS assay used for mutation detection was carried out with a Biocartis Idylla™ system [39]. This in vitro assay is used for the qualitative detection of KRAS (codons 12, 13, 59, 61, 117, and 146). Idylla™ RAS mutation assays use FFPE tumour tissue samples. DNA is firstly extracted and used for gene amplification, followed by real-time PCR detection of the gene of interest. KRAS sequence variations were identified using the Idylla^TM^ molecular diagnostic platform (Biocartis, Mechelen, Belgium). This is a fully automated system that provides sample-to-result technology, being based on the amplification of specific alleles by real-time PCR. The detection limits were a minimum of 25 mm^2^ FFPE tissue for a 5–10 µm slide and a neoplastic cell count >10%. The Idylla™ system is fully integrated with PCR thermocycling and fluorescence detection of target sequences with disposable cartridges. Only two results were inconclusive from the total of 108 analysed cases, requiring repeated testing, probably due to excess paraffin, as mentioned in the manufacturers’ protocol.

### 2.4. Statistical Analysis

Statistical data were calculated using SPSS version 29.0 software. Mean and standard deviation (SD) were calculated for continuous variables, while absolute values and percentages were calculated for categorical variables. The group comparisons were obtained using the Chi-squared test (χ2) and the Fisher correction if necessary. Odds ratio (OD) was calculated to evaluate the association between *KRAS* and tumours’ clinicopathological features, in cases with positive values of all the variables. Comparisons were considered statistically significant when *p* values were <0.05.

## 3. Results

### 3.1. Clinicopathological Features

The clinicopathological features of the patients evaluated in our study, including the demographical profile, the tumour location, and the main microscopic criteria of the histopathological diagnosis have been registered, being summarized in Table 1.

The study group included 108 patients, with 64 men (59.3%) and 44 women (40.7%). The mean age was 64 years (range 24–85 years), with ±13.074 SD and 62 patients (57.4%) being older than the mean age. Regarding the tumour location, 38 tumours (35.2%) were located in the descending/left colon, two tumours (1.9%) were located in the transverse colon, 34 tumours (31.5%) were located in the right colon, and 34 tumours (31.5%) were located in the rectum.

Regarding CRC histological subtype, the most common was adenocarcinoma NOS, diagnosed in 102 cases (94.4%), classified as low-grade in 92 cases (90.2%) and high-grade in 10 cases (9.8%), while the least common subtype was mucinous adenocarcinoma, diagnosed in only six cases (5.6%). A total of 35 tumours (32.4%) showed a tubular histological pattern and 73 tumours (67.6%) showed a mixed histological pattern, with 65 of them (60.2%) exhibiting a glandular and cribriform pattern and 8 of them (7.4%) exhibiting a cribriform and solid pattern.

According to the tumour extension, lymph node involvement, and metastasis, ten tumours (9.8%) were classified as T2, 98 tumours (90.8%) were classified as T3–T4, 10 cases (9.3%) were classified as Nx, 26 cases (24.1%) were classified as N0, 26 cases (24.1%) were classified as N1, and 46 cases (42.6%) were classified as N2, while four patients (3.7%) were included in the metastatic group.

Venous invasion was observed in 66 tumours (61.1%), lymphatic invasion in 88 tumours (81.5%), and perineural invasion in 44 tumours (40.7%). The tumour invasion front was infiltrative in 96 cases (88.9%) and compressive in 12 cases (11.1%). The tumours had an equal distribution of each category of TB (each 33.3%), with more than six tumour buds registered in 58 tumours (53.7%).

### 3.2. KRAS-Mutated Genotype Profile and Its Relationship with Clinicopathological Features

**Figure 1:**
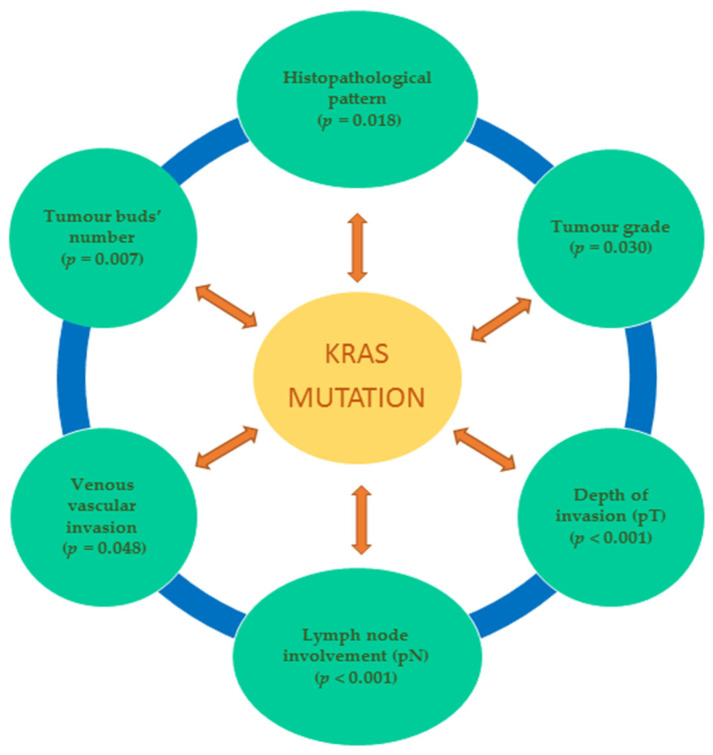
Statistically significant relationships between *KRAS*-mutated genotype and clinicopathological characteristics.

The main clinicopathological features have been analysed according to their KRAS status. A summary of the main clinicopathological features of CRC and their mutated or wild-type KRAS status are shown in Table 2 and Figure 1.

The genetic testing identified the presence of *KRAS* mutation in 54 patients (50%), with 48.1% of *KRAS* mutations identified in women (26 of 44 cases), while 51.9% of *KRAS* mutations identified in men (28 of 64 cases). Regarding the patients’ age, *KRAS* mutation detection was associated with older patients, with only 20 of 54 patients (37.0%) with *KRAS* mutation being younger than the group’s mean age and 34 patients (63.0%) being older than the group’s mean age.

According to the tumours’ location, *KRAS* mutations were equally distributed within the locations, with 33.3% of cases identified in the right colon, 33.3% of cases in the left colon, and 33.3% of cases in the rectum, while no mutations have been identified in the transverse colon tumours.

The analysis of the histological type revealed that *KRAS* mutation was mainly associated with colon adenocarcinoma NOS, as it was detected in 50 of 54 tumours with mutated genotype included in the study (92.6%), while the other four tumours (7.4%) were diagnosed as mucinous type.

According to the histological pattern, *KRAS* mutations were most frequently found in the tubular and cribriform patterns, being detected in 37 cases (68.5%), followed by tubular/glandular patterns, which were detected in 11 cases (20.4%), and cribriform and solid patterns, which were detected in six cases (11.1%).

Considering the tumours’ differentiation, a higher frequency of well- and moderately differentiated tumours was registered. *KRAS* mutation was associated with low-grade tumours in 50 of the mutated cases (92.6%), while the other four cases were diagnosed as high-grade tumours (7.4%). In relation to the tumour extension (T), *KRAS* mutation was mostly associated with T3, as it was detected in 36 T3 tumours (66.7%), followed by T4, as it was detected in 10 T4 tumours (18.5%), and T2, as it was detected in 8 T2 tumours (14.8%).

The lymph node metastases analysis showed an association of *KRAS* mutation with N2, considering that 30 tumours with *KRAS* mutation (55.6%) were included in this category, while 4 mCRCs were not associated with *KRAS* mutation.

According to the lymphovascular invasion, it was detected in 44 cases (81.5%), while 38 cases (70.4%) were associated with venous vascular invasion. A total of 50 cases (92.6%) were associated with an infiltrative pattern of the tumour invasion front. The analysis of TB showed that there was an association between the presence of KRAS mutation and an increased number of tumour buds, with 36 cases (66.7%) containing between 7–12 tumour buds. KRAS mutation was associated with NRAS mutation in two cases (1.85%), without any statistical significance in our study group (Supplementary Files, Tables S1 and S2).

A comparative statistical analysis between the *KRAS* mutation status and tumour clinicopathological features revealed significant associations between *KRAS* mutation and mixed tumour histology (*p* = 0.018), tumour grade (*p* = 0.030), tumour invasion depth (pT) (*p* < 0.001), advanced stage or N2 lymph node metastasis (*p* < 0.001), venous vascular invasion (*p* = 0.048), and high index of tumour budding (*p* = 0.007) (Table 2 and Table 3).

An OR calculation allowed for the identification of *KRAS* mutation association with specific clinicopathological features, as follows: histological cribriform pattern [*p* = 0.018; OR: 3.127 (1.333 ÷ 7.335)], N2 lymph node involvement [*p* < 0.001; OR: 2.969 (1.343 ÷ 6.563)], venous vascular invasion [*p* = 0.048; OR: 2.205 (1.000 ÷ 4.866)], and 7–12 tumour buds [*p* = 0.007; OR: 2. 909 (1.328 ÷ 6.372)], without a statistically significant association between *KRAS* mutation and other clinicopathological characteristics, such as high tumour grade [*p* = 0.030; OR: 0.280 (0.084 ÷ 0.933)] and pT4 invasion depth [*p* < 0.001); OR: 0.211 (0.088 ÷ 0.504)] (Table 2 and Table 3).

## 4. Discussion

CRC is a common neoplasia with an unfavourable prognosis despite therapy progressing, possibly related to the numerous comorbidities of CRC patients, such as disturbances of lipoprotein and carbohydrate metabolisms, associated with cerebrovascular diseases, atherosclerosis, and heart or renal failure [40,41]. Moreover, the association between CRC and diabetes mellitus has been shown to lead to increased resistance to neoadjuvant radiochemotherapy [40,42]. Additionally, the aggressive tumour behaviour may be related to tumour heterogeneity, involving multiple gene alterations and different pathogenic molecular pathways [5], leading to continuous efforts to identify the mechanisms involved in CRC pathogeny and the specific spectrum of gene mutations [11].

It is known that *RAS* mutation is an early event during CRC development [6,43]. There is now solid evidence that *KRAS* mutations are quite common and their worldwide frequency is 40–60% in CRC [6,8,9,44,45], with variations depending on the detection techniques, amount of cases included in the study groups or to the population characteristics. For example, the *KRAS* mutation frequency of occurrence is 37% in the Middle East, with a wide range, between 6.3% and 33.6%, in Iraq [14]; 49.5% in Southern Russia and Nord Caucasus [21]; and 36.5% to 52.7% in China [46]. *KRAS* mutation was also identified in 50% of cases of our study group, complying with the range reported in the literature.

The association between *KRAS* and *NRAS* gene isoforms has been reported in CRC. Moreover, mutations of NRAS codons 12, 13, 61, and 146 lead to similar effects as *KRAS* activation [14]. Although 1.85% of cases investigated in our study showed both *KRAS* and *NRAS* mutations, this association may be validated in larger study groups.

The research review identified only a few studies regarding the frequency of *KRAS* mutations in Romanian patients, including in the North-East region of our country, reporting a range between 39.11% and 83.05% [31,32,33,34,35,36]. Therefore, the population-related data are rather inconsistent in our geographical area, alongside the lack of a centralized national cancer registry, leading to limitations of the statistical evaluation.

The majority of samples in our study (96.29%) were derived from primary tumours, possibly due to the lack of systematic metastases biopsy or to the rapid progression of the disease, in agreement with the findings of other studies [4,43].

CRC may occur in patients of all ages, as demonstrated by the wide age range in the study group, between 24 and 85 years, with a mean age of 64 years, while 57.4% of patients were older than the group average, with a predominant distribution for men (59.3%), a profile in agreement with the literature findings [4,6,47]. The data analysis revealed some general characteristics of the patients in the study group, as follows: tumours located in the left colon (35.2%), followed by an equal distribution of tumours located in the right colon (31.5%) and in the rectum (31.5%), mostly showing adenocarcinoma NOS-type histology (94.4%), and low-grade pattern (85.2%).

*KRAS* mutations were identified more frequently in older patients than the average group age, but without a statistically significant registered relationship, in contrast to the literature data [4]. These differences could be attributed to different diagnostic techniques, genetic factors, and geographical distributions.

Although the tumour location is considered a predictive and prognostic factor for CRC treatment response [48], there are conflicting results regarding the association between *KRAS* mutations and tumour location. *KRAS* gene mutations seem to be more common in tumours located in the right colon [4,49] or in the descending colon [5], in different studies.

The rate of rectal cancer increased worldwide from 27% to 31%, between 1995 and 2019 [50], being also revealed in our study, which showed a left colon distribution in 35.2%, along with an equal rate of right colon and rectal tumours (each 31.5%). Regarding the association with *KRAS* mutation, an equal distribution was detected for the right colon, left colon, and rectum, with a 33.3% detection rate for any location.

The literature data support a predominant adenocarcinoma NOS type [6], in agreement with our study group, with only six cases (5.55%) diagnosed as a mucinous type. A mixed histological pattern (tubular and cribriform) was observed in more than a half of cases (60.2%), with T3 in 55.6% of cases, with N2 in 42.6% of cases, but with very rare distant metastases (3.7%), with a predominantly infiltrative invasion front (88.88%), with more than half of the tumours (53.7%) having 7–12 tumour buds, corresponding to CRC heterogeneity.

Among 104 patients (96.3%) with M0 stage, 54 patients (51.9%) had *KRAS* gene mutation, in agreement with the literature data [6,32], without any statistically significant relationship between the detection of distant metastasis and the frequency of *KRAS* mutations, in our study.

The analysis of the venous invasion showed its detection in 66 cases (61.1%), with 38 of these cases (70.4%) harbouring *KRAS* gene mutations, with a statistically significant association between *KRAS* mutation status and venous invasion (*p* = 0.048), in our study group. This finding is different from data reported by other research teams that identified a rather high percentage of cases with *KRAS* gene mutation (73.5%), in the absence of venous invasion [6]. This feature may be related to *RAS* gene mutation occurrence in the early stages of disease development [6,43], prior to the venous invasion. However, our study group results may correspond to more advanced stages in CRC progression.

Although the molecular mechanisms underlying TB are not completely deciphered, evidence suggests an association between TB and specific gene alterations, including *KRAS* mutations [51]. The comparative analysis showed a statistically significant association between the number of tumour clusters and *KRAS* mutation (*p* = 0.007) in our study group. Additionally, TB has the value of an independent prognostic biomarker in a variety of solid tumours, including CRC [51,52,53,54], being related to tumour progression [52,53,54]. Despite some evidence suggesting an association between *KRAS* mutations and TB in CRC, further research is needed to assess its clinical implications [51,52].

The frequency of *KRAS* mutation in our study group complies with the literature data. A comparative statistical analysis revealed significant associations between *KRAS* mutation and some clinicopathological features, such as tumour histological pattern (*p* = 0.018), tumour grade (*p* = 0.030) depth of invasion (pT) (*p* < 0.001), lymph node involvement (pN) (*p* < 0.001), and tumour budding (*p =* 0.007). Additionally, OR calculation showed that *KRAS* mutation showed a strong association with cribriform tumour histology, N2 lymph node involvement (pN), venous invasion, and a high number of tumour buds (between 7 and 12). Considering that these clinicopathological features are associated with an unfavourable prognosis, our results may suggest that the detection of *KRAS* mutation may be an indicator of unfavourable outcome, a finding which is consistent with the results of other studies [6,51]. However, our findings need to be validated in more extensive study groups.

The association between *KRAS* mutation and unfavourable clinicopathological findings is not surprising, as the classically activated *KRAS* gene, through activation of receptor tyrosine kinase kinases (RTKs) by their binding to various growth factors, triggers dimerization and transphosphorylation processes that regulate different critical cellular processes [55,56,57,58]. Furthermore, *KRAS* acts as a signalling hub mediating several downstream signalling molecular pathways, such as serine/threonine kinase Raf-mitogen activated protein kinase-extracellular regulated kinase (RAF-MEK-ERK), phosphoinositide 3-kinases-protein kinase B-mammalian target of rapamycin (PI3K-AKT-mTOR), Rac-specific guanine nucleotide exchange factor Tiam1 (TIAM1-RAC), and Ral guanine nucleotide dissociation stimulator (RalGDS-Ral) [57,58,59]. Therefore, *KRAS* mutations activation is involved in carcinogenesis by the activation of multiple downstream signalling pathways.

Despite considerable efforts to target KRAS mutations, modest success has been achieved due to the versatility of the structural conformation induced by mutations and the amount of upstream and downstream regulatory pathways, which can induce the development of tumours with multiple mechanisms of resistance to new therapies [8,19,56,60,61].

The literature data from the recent decade highlight the roles of tumour-associated mast cells, microRNAs (miRNAs), and *BRAF*, alongside *KRAS* genes, in CRC initiation and progression [62]. Moreover, approximately 15% of CRC patients have microsatellite instability (MSI), which can be classified into three groups according to the frequency of MSI: microsatellite-stable (MSS), low-frequency MSI (MSI-L), and high-frequency MSI (MSI-H) [63]. Accordingly, the accurate determination of MSI status is essential for CRC treatment strategy approaches, and MSI testing is currently recommended for all CRC patients [22,63,64]. MSI is known to occur in CRC associated with Lynch syndrome as well as in sporadic cancers subsets [24]. In addition, tumours with microsatellite instability are sensitive to immune checkpoint inhibitors [63,64]. Moreover, a *BRAF* mutation is associated with MSI through *BRAF* mutation’s relationship with high-level CpG island methylator phenotype (CIMP) and MLH1 promoter methylation [14], with significant implications for disease aggressiveness and clinical management [30].

Long-course chemoradiotherapy (LCRT) and short-course radiotherapy (SCRT), as neoadjuvant therapies, may lead to improved local control of locally advanced disease [65,66]. Supplementarily, immune checkpoint inhibitor therapy shows promising results, especially in mismatch repair-deficient (dMMR) or MSI-H tumours [65]. The classification of patients into different groups based on their genetic and epigenetic characteristics and their susceptibility to a specific disease is useful for the selection of the best therapeutic approach [67], including *RAS* mutations, especially in the context of increasing resistance to monoclonal antibody therapy, mainly applied in mCRC [15,68]. Accordingly, the current necessity of genetic analyses on a larger scale, which requires the implementation of a Romanian national program, is strongly supported by our study.

Our results add to the large number of mainstream studies focusing on the involvement of *KRAS* mutations in CRC diagnose and treatment. Considering the reduced number of studies conducted on patients in Romania, including the North-East region, our study provides significant data, which confirm the frequency of *KRAS* mutation occurrence in CRC patients. However, our study had several limitations, such as (i) the small number of samples available for genetic tests; (ii) the lack of data on all potential prognostic factors, such as MSI, *BRAF*, PIK3CA, *TP53*, *APC*, mast cells, comorbidities, lifestyle factors, and socioeconomic status; (iii) the lack of survival and recurrence data; and (iv) the limited statistical significance for *NRAS* mutation tests.

Despite these limitations, the objectives of our study to identify potential challenges and refine the research protocols prior to more extensive investigations have been met.

## 5. Conclusions

Our findings demonstrate an association between *KRAS* mutations and specific clinicopathological features, such as tumour histological pattern, tumour grade, depth of invasion (pT), nodal involvement (pN), venous vascular invasion, and tumour bud number, which are important parameters for tumour progression and management. However, our results could not evaluate *NRAS* mutations’ signification due to their reduced amount, requiring an extensive study group for future validation.

Our results are providing new perspectives for breakthrough research in the context of an envisaged increase in accessibility for CRC patients to genetic testing. Future implementations of population-based screening programmes in our region would be useful for the selection of patients eligible for targeted therapy in the context of personalized cancer therapeutic approach.

## Figures and Tables

**Table 1:** Clinicopathological characteristics of the patients.

Clinicopathological Characteristics	No. of Cases	Percentage (%)
Gender		
Women	44	40.7
Men	64	59.3
**Age**		
Age (Mean ± SD)	64 ± 13.074	
≤64 years	46	42.6
>64 years	62	57.4
Tumour location		
Right colon	34	31.5
Transverse colon	2	1.9
Left colon	38	35.2
Rectum	34	31.5
Histopathological type		
Adenocarcinoma NOS	102	94.4
Mucinous adenocarcinoma	6	5.6
Histological pattern		
Tubular/glandular	35	32.4
Cribriform and solid	8	7.4
Tubular and cribriform	65	60.2
Grade		
High	16	14.8
Low	92	85.2
Depth of invasion (pT)		
T2	10	9.8
T3	60	55.6
T4	38	35.2
Lymph node involvement (pN)		
Nx	10	9.3
N0	26	24.1
N1	26	24.1
N2	46	42.6
Distant metastasis (pM)		
M0	104	96.3
M1	4	3.7
Lymphovascular invasion		
Present	88	81.5
Absent	20	18.5
Venous vascular invasion		
Present	66	61.1
Absent	42	38.9
Perineural invasion		
Present	44	40.7
Absent	64	59.3
Tumour invasion front type		
Infiltrative	96	88.9
Compressive	12	11.1
Tumour budding (TB)		
BD1	36	33.3
BD2	36	33.3
BD3	36	33.3
Tumour buds number range		
3–6	50	46.3
7–12	58	53.7

BD1—“low budding”, BD2—“intermediate budding”, BD3—“high budding”, No.—“number”, NOS—“not otherwise specified”, SD—“standard deviation”.

**Table 2:** Relationship between KRAS genotype status and clinicopathological features.

	*KRAS* Genotype		
Clinicopathological Characteristics	Mutated No./%	Wild-Type No./%	*p* Value ^†^
Gender			
Women	26/48.1	18/33.3	0.117
Men	28/51.9	36/66.7	
Age group			
≤64 years	20/37.0	26/48.1	0.243
>64 years	34/63.0	28/51.9	
Tumour location			
Right colon	18/33.3	16/29.6	0.505
Transverse colon	0/0	2/3.7	
Left colon	18/33.3	20/37.0	
Rectum	18/33.3	16/29.6	
Histopathological type			
Adenocarcinoma NOS	50/92.6	52/96.3	0.678
Mucinous adenocarcinoma	4/7.4	2/3.7	
Histological pattern			
Tubular/glandular	11/20.4	24/44.4	0.018 *
Cribriform and solid	6/11.1	2/3.7	
Tubular and cribriform	37/68.5	28/51.9	
Grade			
High	4/7.4	12/22.2	0.030 *
Low	50/92.6	42/77.8	
Depth of invasion (pT)			
T2	8/14.8	2/3.7	<0.001 *
T3	36/66.7	24/44.4	
T4	10/18.5	28/51.9	
Lymph node involvement (pN)			
Nx	8/14.8	2/3.7	<0.001 *
N0	10/18.5	16/29.6	
N1	6/11.1	20/37.0	
N2	30/55.6	16/29.6	
Distant metastasis (pM)			
M0	54/100	50/92.6	0.118
M1	0/0	4/7.4	
Lymphovascular invasion			
Present	44/81.5	44/81.5	1.000
Absent	10/18.5	10/18.5	
Venous vascular invasion			
Present	38/70.4	28/51.9	0.048 *
Absent	16/29.6	26/48.1	
Perineural invasion			
Present	24/44.4	20/37.0	0.433
Absent	30/55.6	34/63.0	
Tumour invasion front type			
Infiltrative	50/92.6	46/85.2	0.221
Compressive	4/7.4	8/14.8	
Tumour budding (TB)			
BD1	18/33.3	18/33.3	0.169
BD2	14/25.9	22/40.7	
BD3	22/40.7	14/25.9	
Tumour buds number			
3–6	18/33.3	32/59.3	0.007 *
7–12	36/66.7	22/40.7	

^†^ Chi-square test; *p* < 0.05 * statistically significant; BD1—“low budding”, BD2—“intermediate budding”, BD3—“high budding”, *KRAS*—“Kirsten rat sarcoma virus”, No.—“number”, NOS—“not otherwise specified”.

**Table 3:** Analysis of KRAS mutation association with specific clinicopathological features.

*KRAS*	OR (95% CI)	*p*-Value ^†^
Histological pattern		
Cribriform vs. other types	OR: 3.127 (1.333 ÷ 7.335)	0.018 *
Grade		
High vs. low	OR: 0.280 (0.084 ÷ 0.933)	0.030 *
Depth of invasion (pT)		
T4 vs. T2 and T3	OR: 0.211 (0.088 ÷ 0.504)	<0.001 *
Lymph node involvement (pN)		
N2 vs. Nx, N0, and N1	OR: 2.969 (1.343 ÷ 6.563)	<0.001 *
Venous vascular invasion		
Present vs. Absent	OR: 2.205 (1.000 ÷ 4.866)	0.048 *
Tumour buds’ number		
3–6 vs. 7–12	OR: 2.909 (1.328 ÷ 6.372)	0.007 *

OR: “odds ratio”; CI: “confidence interval” ^†^ Chi-square test; *p* < 0.05 * statistically significant; BD1—“low budding”, BD2—“intermediate budding”, BD3—“high budding”, *KRAS*—“Kirsten rat sarcoma virus”.

## Data Availability

The data used to support the findings of this research are available upon request to the authors.

## Supplementary Materials

The following supporting information can be downloaded at https://www.mdpi.com/article/10.3390/cimb47020120/s1.
