# Supplementary material for: KRAS Mutation Status in Relation to Clinicopathological Characteristics of Romanian Colorectal Cancer Patients
Source: Curr Issues Mol Biol. 2025 Feb 12;47(2):120. doi: 10.3390/cimb47020120 (PMC11854687; doi:10.3390/cimb47020120)
Supplement: Supplementary file 1 [file cimb-47-00120-s001.zip › cimb-3348659-supplementary.pdf]

Table S1. Relationship between NRAS status and clinicopathological features.

	NRAS Genotype		
**Clinicopathological characteristics**	**MUTATED No./%**	**WILD-TYPE No./%**	***p* value†**
**Gender**			
Female	0/0.0	44/42.3	0.144
Male	4/100.0	60/57.7	
**Age group**			
≤ 64 years	2/50.0	44/42.3	1.000
> 64 years	2/50.0	60/57.7	
**Tumour location**			
Right colon	0/0.0	34/32.7	0.553
Transverse colon	0/0.0	2/1.9	
Left colon	2/50.0	36/34.6	
Rectum	2/50.0	32/30.8	
**Histopathological type**			
Adenocarcinoma NOS	2/50.0	100/96.2	**0.015***
Mucinous adenocarcinoma	2/50.0	4/3.8	
**Histopathological pattern**			
Tubular/glandular	4/100.0	31/29.8	**0.013***
Cribriform and solid	0/0.0	8/7.7	
Tubular and cribriform	0/0.0	65/62.5	
**Grade**			
High	2/50.0	14/13.5	0.104
Low	2/50.0	90/86.5	
**Depth of invasion (pT)**			
T2	0/0.0	10/9.6	0.190
T3	4/100.0	56/53.8	
T4	0/0.0	38/36.5	
**Lymph node involvement (pN)**			
Nx	2/50.0	8/7.7	**0.023***
N0	0/0.0	26/25.0	
N1	0/0.0	26/25.0	
N2	2/50.0	44/42.3	
**Distant metastasis (pM)**			
M0	4/100.0	100/96.2	1.000
M1	0/0.0	4/3.8	
**Lymphovascular invasion**			
Present	4/100.0	84/80.8	1.000
Absent	0/0.0	20/19.2	
**Venous vascular invasion**			
Present	4/100.0	62/59.6	0.155
Absent	0/0.0	42/40.4	

**Perineural invasion**			
Present	2/50.0	42/40.4	
Absent	2/50.0	62/59.6	1.000
**Tumour invasion front**			
Infiltrative	4/100.0	92/88.5	1.000
Compressive	0/0.0	12/11.5	
**Tumour budding (TB)**			
BD1	2/50.0	34/32.7	
BD2	0/0.0	36/34.6	0.354
BD3	2/50.0	34/32.7	
**Tumour buds number**			
3-6	2/50.0	48/46.2	1.000
7-12	2/50.0	56/53.8	

†Chi-square test; *p* < 0.05* statistically significant; BD1 - low budding, BD2 – intermediate budding, BD3 – high budding, NRAS - Neuroblastoma RAS viral oncogene homolog.

Table S2. Analysis of NRAS mutation as risk factor for tumor's clinicopathological features

NRAS	OR (95% CI)	p -value
**Histopathological type**		
Adenocarcinoma NOS	OR: 25.000 (2.770÷225.638)	**0.015***
Mucinous adenocarcinoma		

OR: odds ratio; CI: confidence interval †Chi-square test; *p* < 0.05* statistically significant; NRAS - Neuroblastoma RAS viral oncogene homolog.

Table S3 KRAS and NRAS mutation frequencies in reported Romanian studies.

	**References**	**Location**	**Study period of time**	**Patients' group number**	**Study subject**	**Material**	**Methods**	**KRAS/NRAS mutation frequencies**
**1**	Negru et al., 2014	Greece and Romania	NA	2071 total 1699 (Greece) 372 (Romania)	CRC	FFPE	Sanger sequencing	41.3%/NA (Greek) 39.2%/NA (Romanian)
				354				40.96/15.31
**2**	Brinzan et al., 2019	S-E Romanian Region	NA	56	CRC	FFPE	StripAssay method based on (PCR) and reverse-hybridization	50%/NA
**3**	Brinzan et al., 2022	S-E Romanian Region	NA	63	NA	Fresh tumor samples	Sanger sequencing	39.63%/0
**4**	Afrăsînei et al.,2023	N-E Romani	2017-2019	104	MTS CRC	FFPE	NGS TruthLight	42.6%/4,8%

		an Region					Tumor, Illumina Platform	
**5**	Pîrvu et al., 2023	S-E Romanian Region	2015-2023	225	MTS CRC	FFPE	NGS Ion AmpliSeq NGS Panel	39.11%/NA
**6**	Vesa et al., 2023	N-W Romanian Region	2018-2022	118	CRC	FFPE	real-time PCR method	83.05%/3.38%

CRC - „Colorectal Carcinoma"; FFPE - „Formalin-Fixed Paraffin-Embedded";-PCR - „polymerase chain reaction" S-E - „South-East"; MTS - „Metastasis"; NA - „ not available"; N-E - „North-East"; NGS - „Next Generation Sequencing"; N-W - „North-West"
